# Nanoparticle Sphericity Investigation of Cu-Al_2_O_3_-H_2_O Hybrid Nanofluid Flows between Inclined Channels Filled with a Porous Medium

**DOI:** 10.3390/nano12152552

**Published:** 2022-07-25

**Authors:** Xiangcheng You

**Affiliations:** State Key Laboratory of Petroleum Resources and Prospecting, China University of Petroleum, Beijing 102249, China; xcyou@cup.edu.cn

**Keywords:** nanoparticle sphericity, Cu-Al_2_O_3_-H_2_O hybrid nanofluid, inclined channel, porous medium

## Abstract

With the porous medium-filling inclined channels, we investigate the nanoparticle sphericity of Cu-Al_2_O_3_-H_2_O hybrid nanofluid flows. We consider the constant flow rate through the channels as well as the uniform heat flux on wall channels. We provide analytical solutions for both the velocity and temperature fields. Several parameters are considered in the analytical solutions, including the mixed convection variable, the Peclet number, the channel tilt angle, and nanoparticle sphericity and volume fractions. The significant findings of this study are that the effective thermal conductivity increases when increasing the temperature in the same nanoparticle volume fractions. Nanoparticles with a smaller average sphericity size have a greater specific surface area and contain a greater concentration of small particles, which enhances the internal heat transfer of nanofluids. The other noteworthy observation of this study is that when the nanoparticle volume fraction increases from 0.1 to 0.2, although the heat transfer enhancement rate has slowed down, it has also increased by about 25%. The hybrid nanofluids have suitable stability, and the enhanced heat transfer effect is better with the increase in nanoparticle compositions.

## 1. Introduction

As is well known, Choi et al. [1] first presented the idea of a nanofluid in 1995. Nanofluids are suspensions formed by adding nanoparticles in a certain way and proportionally to the base liquid, such as ethanediol, fuel oil, or water. In order to improve the positive characteristics of conventional nanofluids, the concept of hybrid nanofluid was proposed, which is formulated by adding two or more nanoparticles with different properties to a base liquid. Many researchers have found hybrid nanofluids to be of great interest, as they have a wide range of industrialized, technical, and mechanical uses, such as aeroacoustics, conveyance, marine structures, microfluidics, clinical lubrication, heat-exchange applications, generator cooling, and petroleum engineering [2,3,4]. How nanotechnology and nanoparticles may be applied to the oil and gas industry has also been widely studied, including in drilling fluid, cementing, oil well stimulation, and enhanced oil recovery. Researchers have studied hybrid nanofluids for a long time, but it is critical that we expand the scope of our research to properly utilize hybrid nanofluids. In the process of its practical application, sometimes, the fluid needs to have several properties at the same time, such as suitable stability, high thermal conductivity, and excellent rheological properties. Mixed nanofluids may meet all of these requirements due to the addition of several nanoparticles with different properties at the same time [5]. Metal nanoparticles have high thermal conductivity but are easily oxidized. At present, Al_2_O_3_, Cr_2_O_3_, and ZrO particles have been added to a copper matrix. Al_2_O_3_ nanoparticles have low production cost, high hardness, and suitable stability but very low thermal conductivity. Therefore, Al_2_O_3_ nanoparticles are the most commonly used reinforcing phase for copper-based materials at present. As Cu particles have a larger particle size compared to Al_2_O_3_ particles, Al_2_O_3_ particles can fill the channel formed by Cu particles to form a tighter nanolayer structure. The random motion of particles caused by Brownian motion and the thermal motion of liquid molecules form a solid–liquid interface nanolayer with lower thermal resistance that enhances heat transfer. However, with an increase in the particle diameter in hybrid nanofluids, the random motion rate decreases, and the nanoparticles form large aggregates. At this time, Brownian motion reduces the heat transfer in nanofluids. Consequently, the sphericity of the hybrid nanofluid particles must be studied closely. Devi et al. [6] conducted a numerical investigation of hydromagnetic Al_2_O_3_-Cu-H_2_O flows over permeable and stretching sheets with suction and observed that hybrid nanofluids had a higher heat conduction rate than nanofluids in magnetic field conditions. Maskeen et al. [7] investigated the heat conduction of hydromagnetic Al_2_O_3_-Cu-H_2_O flows over stretching cylinders affected by Lorentz magnetic force and thermic emission. Wainia et al. [8] investigated the steady flows of hybrid nanofluids through permeable moving surfaces, which solved the similarity equations numerically, and found that hybrid nanofluids enhanced heat conduction compared to conventional nanofluids. Elsaid et al. [9] studied mixed convection hybrid nanofluids in vertical channels with the effects of thermic emission. The presence of thermic radiation improved heat conduction from the base liquid by 12% to 22%, depending on the ratio of hybrid nanoparticles. Alazwari et al. [10] examined the entropy production as thermodynamically stable first-grade viscoelastic nanofluid (FGVNF) flow over a flat, penetrable, porous barrier. Nanofluids had better surface stability and thermal absorption, and distribution capacities were produced as heat transfer fluids. Waqas et al. [11] investigated the Darcy–Forchheimer flow of Reiner–Philippoff nanofluids with a heat source/sink and noted thermal conductivity with the occurrence of motile microorganisms over the stretching surface. Nanofluids are also more practical for enhancing heat transfer compared to regular fluids. Jamshed et al. [12] investigated the unsteady flow of a non-Newtonian Casson nanofluid in terms of its thermal transport as well as entropy. The impact of the slip condition and solar thermal transport in terms of convection regarding Casson nanofluid flow were investigated thoroughly. Rashidi et al. [13] reviewed the features of nanofluids with hybrid nanostructures and proposed models for these properties. It was concluded that the increase in the volume fraction of solids caused an improvement in thermal conductivity and dynamic viscosity, while the trend of variations in the specific heat depended on the base fluid. Nonlaopon et al. [14] investigated the heat transfer of two-phase nanofluid flow between horizontal plates in a rotating system with a magnetic field and external forces. An efficient stochastic technique based on feed-forward neural networks (FFNNs) with a back-propagated Levenberg–Marquardt (BLM) algorithm was developed to examine the effect of variations in various parameters on velocity, gravitational acceleration, temperature, and concentration profiles of the nanofluid. Dero et al. [15] investigated mathematical modeling using a Tiwari and Das nanofluid model, taking into account the effects of magnetic, suction/injection, and thermal radiation, as well as the stability analysis of a hybrid nanofluid containing copper and alumina nanoparticles in a water-based liquid. Mannu et al. [16] presented the first report on the drug loading/release capability of MNF formulated with methoxy polyethylene glycol (referred to as PEG)-coated MNP in an aqueous (phosphate buffer) fluid. Magnetic nanoparticles (MNPs) are widely used materials for biomedical applications due to their intriguing chemical, biological, and magnetic properties. The evolution of MNP-based biomedical applications (such as hyperthermia treatment and drug delivery) could be advanced using magnetic nanofluids (MNFs) designed with a biocompatible surface-coating strategy.

There are many pieces of literature that indicate that the nanoparticle shape has a considerable influence on the nanofluid’s thermal conductivity. Numerical simulations of natural convection flow and heat transfer in a trapezoidal enclosure filled with different types of nanofluids were carried out by Sheikha et al. [17]. Ho et al. [18] used experimental and numerical methods to study convection heat transfer through a round pipeline containing Al_2_O_3_-water nanofluids. It was found that nanofluids can not only reduce the wall temperature but also enhance heat transfer. Wang et al. [19] provided numerical methods to investigate variations in the physical parameters affecting the forced convection heat transfer by Al_2_O_3_-water nanofluids in microchannels. Research in the past has mostly concentrated on fluid flow in horizontal and vertical channels [20,21,22,23,24]. Although many researchers have worked on hybrid nanofluids [25,26,27,28], there are relatively few studies on mixed convection in inclined geometries, and these models hardly consider how nanoparticle shape affects hybrid nanofluids flow and heat transfer [29,30]. Lavine [31] described how to develop laminar flow between inclined parallel plates. The velocity dissipated by laminar mixed convection in inclined channels under certain temperature conditions was studied by Barletta et al. [32]. Flows of mixed convective heat conduction of magnetic fluid on tilted plates were investigated by Aidin et al. [33]. Cimpean [34] examined mixed convective flows of nanofluids in tilted channels filled with porous media. As part of a numerical study, Goyal et al. [35] investigated the flow of nanofluids through an inclined heated plate under the influence of a magnetic field. By increasing the tilt angle parameter, the thermal boundary-layer thickness is increased. Khademi et al. [36] employed numerical methods to study how the mixed convective flow of nanofluids on inclined plates in porous media is affected by transverse magnetic fields. A study of convective heat conduction in nanofluids whose walls are heated by uniform heat flux between inclined channels was performed by You et al. [37,38]. Anuar et al. [39] investigated heat conduction and boundary-layer flows of hybrid nanofluids using inclined stretch/shrink thin plates, as well as the suction and buoyancy effects. In this paper, except for the nanoparticle volume fraction, the influence of nanoparticle sphericity on mixed convective flows and the heat conduction of hybrid nanofluids between inclined channels filled with porous media are studied. In addition, the flow structure and thermic transport are analyzed in relation to the nanoparticle volume fraction and nanoparticle sphericity.

## 2. Model of Mathematics

**Figure 1:**
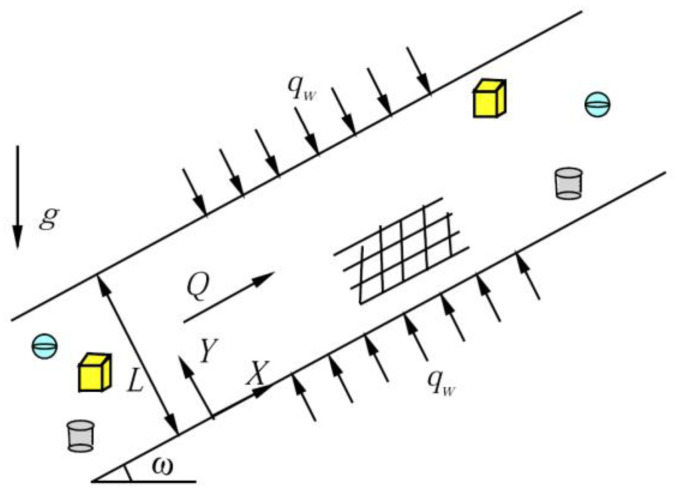
Coordinates for the physical configuration.

An external gradient in pressure and buoyancy may drive mixed convection in steady-state conditions. The pressure gradient is located between two parallel inclined plates filled with a hybrid nanofluid, and the separation distance is L. Coordinates for the physical configuration are shown in Figure 1. The X axis follows the bottom plate, the Y axis is perpendicular to it, g represents gravity acceleration, $q_{w}$ is constant heat flux, and ω is the inclined angle of the inclined channel. The hybrid nanofluid containing different nanoparticles is filled between inclined channels. Defining Darcy’s law with Boussinesq’s approximation and hybrid nanofluid models as references [31,34], the momentum balance equations and the energy equations are:

(1) $$\frac{\partial U}{\partial X}+\frac{\partial V}{\partial Y}=0,$$

(2) $$\frac{\mu_{eff}}{K}\left(\frac{\partial U}{\partial Y}-\frac{\partial V}{\partial X}\right)={\left(\rho \beta \right)}_{hnf}g\left(\frac{\partial T}{\partial Y}\sin\omega -\frac{\partial T}{\partial X}\cos\omega \right)$$

(3) $$U\frac{\partial T}{\partial X}+V\frac{\partial T}{\partial Y}=\alpha_{m}\left(\frac{\partial^{2}T}{\partial X^{2}}+\frac{\partial^{2}T}{\partial Y^{2}}\right)$$

subject to defined boundaries:

(4) $$U\left(0\right)=0\begin{array}{cc} , & - \end{array}{\left.\frac{\partial T}{\partial Y}\right|}_{Y=0}=1$$

(5) $$U\left(L\right)=0\begin{array}{cc} , & - \end{array}{\left.\frac{\partial T}{\partial Y}\right|}_{Y=L}=\frac{q_{w}}{k_{f}}$$

This channel flow analysis assumes mass flow rate as a predetermined quantity, so we must determine this section’s average fluid velocity as follows:

(6) $$Q=\int_{0}^{L}U\left(Y\right)dY$$

where T is the temperature of hybrid nanofluids, Q represents the average speed of hybrid nanofluid, K is porous medium’s permeability, $\alpha_{m}$ is effective thermal diffusivity, and $\mu_{eff}$ represents effective viscosity, whose value is determined by porous media structure and flow strength, $k_{hnf}$ is the thermal conductivity of hybrid nanofluids, and ${\left(\rho \beta \right)}_{hnf}$ represents density and thermic expansion of hybrid nanofluid.

It appears that the continuity equation is simplified to ∂U/∂X and the velocity field is reduced to $V\left(U,0\right)$, which can be written as $U=U\left(Y\right)$. Consequently, Equations (1)–(3) become:

(7) $$\frac{\mu_{hnf}}{\mu_{f}}\frac{\partial U}{\partial Y}={\left(\rho \beta \right)}_{hnf}\frac{gK}{\mu_{f}}\left(\frac{\partial T}{\partial Y}\sin\omega -\frac{\partial T}{\partial X}\cos\omega \right)$$

(8) $$U\frac{\partial T}{\partial X}=\alpha_{hnf}\frac{\partial^{2}T}{\partial Y^{2}}$$

Using dimensionless parameters:

(9) $$x=\frac{X}{L}\begin{array}{cc} , & y=\frac{Y}{L} \end{array}\begin{array}{cc} , & u\left(y\right) \end{array}=\frac{U}{U_{0}}\begin{array}{cc} , & \theta \left(x,y\right) \end{array}=\frac{k_{f}\left(T-T_{0}\right)}{q_{w}L}$$

where $U_{0}=Q/L$ is reference velocity and $T_{0}$ is inflow fluid temperature. Substituting Equation (9) into Equations (7) and (8), we can obtain:

(10) $$\frac{\partial u}{\partial y}=\lambda {\left(1-\varphi_{1}\right)}^{2.5}{\left(1-\varphi_{2}\right)}^{2.5}\frac{{\left(\rho \beta \right)}_{hnf}}{{\left(\rho \beta \right)}_{f}}\left(\frac{\partial \theta }{\partial y}\sin\omega -\frac{\partial \theta }{\partial x}\cos\omega \right)$$

(11) $$\mathrm{Pe}u\frac{\partial \theta }{\partial x}=\frac{\alpha_{hnf}}{\alpha_{f}}\frac{\partial^{2}\theta }{\partial y^{2}}$$

subject to defined boundaries:

(12) $$u\left(0\right)=0\begin{array}{cc} , & - \end{array}{\left.\frac{\partial \theta }{\partial y}\right|}_{y=0}=1$$

(13) $$u\left(1\right)=0\begin{array}{cc} , & - \end{array}{\left.\frac{\partial \theta }{\partial y}\right|}_{y=1}=1$$

as well as mass flux conservation:

(14) $$\int_{0}^{1}udy=1$$

Here, $\lambda ={\left(\rho \beta \right)}_{f}gKqL/\left(U_{0}\mu_{f}k_{f}\right)$ is the mixed convection variable and $\mathrm{Pe}=U_{0}L/\alpha_{f}$ is the Peclet number. We can assume Pe>0 and consider that the upward inclined channel with the range of tilt angle is limited to 0<ω<π/2. We do not consider the special cases of horizontal (ω=0) and vertical (ω=π/2) conditions in this paper.

We can assume that water base fluid and nanoparticles in hybrid nanofluids are in thermal equilibrium and have no relative slip velocity. The hybrid nanofluid is incompressible and mixed convective between two parallel inclined plates. Table 1 shows the thermal characters of water base fluid and nanoparticles [40,41]. The effective density, specific heat capacity, dynamic viscosity, thermal diffusivity, and thermal expansion coefficients of hybrid nanofluids [42,43,44] are calculated by the following formula:

(15) $$\begin{array}{l} \rho_{hnf}=(1-\varphi_{2})[\rho_{f}(1-\varphi_{1})+\rho_{n1}\varphi_{1}]+\rho_{n2}\varphi_{2},\\ {\left(\rho C_{p}\right)}_{hnf}=(1-\varphi_{2})[{\left(\rho C_{p}\right)}_{f}\left(1-\varphi_{1}\right)+{\left(\rho C_{p}\right)}_{n1}\varphi_{1}]+{\left(\rho C_{p}\right)}_{n2}\varphi_{2},\\ \alpha_{hnf}=\frac{k_{hnf}}{{\left(\rho C_{p}\right)}_{hnf}}\begin{array}{cc} , & \mu_{hnf} \end{array}=\frac{\mu_{f}}{{\left(1-\varphi_{1}\right)}^{2.5}{\left(1-\varphi_{2}\right)}^{2.5}},\\ {\left(\rho \beta \right)}_{hnf}=(1-\varphi_{2})[{\left(\rho \beta \right)}_{f}\left(1-\varphi_{1}\right)+{\left(\rho \beta \right)}_{n1}\varphi_{1}]+{\left(\rho \beta \right)}_{n2}\varphi_{2}. \end{array}$$

In order to calculate hybrid nanofluid’s thermal conductivity, we use the formula proposed by [45]:

(16) $$\begin{array}{l} \frac{k_{hnf}}{k_{nf}}=\frac{k_{n2}-\varphi_{2}(s-1)(k_{nf}-k_{n2})+k_{nf}(s-1)}{k_{n2}+\varphi_{2}(k_{nf}-k_{n2})+k_{nf}(s-1)},\\ \frac{k_{nf}}{k_{f}}=\frac{k_{n1}-\varphi_{1}(s-1)(k_{f}-k_{n1})+k_{f}(s-1)}{k_{n1}+\varphi_{1}(k_{f}-k_{n1})+k_{f}(s-1)}. \end{array}$$

where the subscript f represents the base liquid, nf represents the nanofluids, hnf represents the hybrid nanofluids, φ represents nanoparticles volume fraction, s is the shape factor of nanoparticles, s=3/ψ, and ψ is nanoparticle sphericity. When the shape of nanoparticles is platelet, cylinders, brick, and spherical [45], and equivalent diameter DP = 45 nm, the sphericity is 0.52, 0.61, 0.81, and 1.00, respectively, as shown in Figure 1.

By considering Equations (7) and (8) based on the reference paper by Cimpean et al. [34,40], a solution is provided:

(17) $$u=u\left(y\right)\begin{array}{cc} , & \theta \left(x,y\right) \end{array}=c_{0}x+t\left(y\right)$$

Substituting Equation (17) into Equation (8) with condition Equation (13), taking into account the channel cross-section:

(18) $$\frac{\alpha_{f}\mathrm{Pe}}{\alpha_{hnf}}c_{0}\int_{0}^{1}u\left(y\right)dy={\left.\frac{\partial t}{\partial y}\right|}_{y=1}-{\left.\frac{\partial t}{\partial y}\right|}_{y=0}=2$$

Substituting Equations (17) and (18) into Equations (10) and (11), they become:

(19) $$\frac{\partial u}{\partial y}=\lambda {\left(1-\varphi_{1}\right)}^{2.5}{\left(1-\varphi_{2}\right)}^{2.5}\frac{{\left(\rho \beta \right)}_{hnf}}{{\left(\rho \beta \right)}_{f}}\left(\frac{d\theta }{dy}\sin\omega -\frac{2\alpha_{hnf}}{\alpha_{f}\mathrm{Pe}}\cos\omega \right)$$

(20) $$2u=\frac{d^{2}\theta }{dy^{2}}$$

Combining Equations (19) and (20), we can obtain a third-order differential equation by following these steps:

(21) $$\frac{d^{3}t}{dy^{3}}-2\lambda {\left(1-\varphi_{1}\right)}^{2.5}{\left(1-\varphi_{2}\right)}^{2.5}\frac{{\left(\rho \beta \right)}_{hnf}}{{\left(\rho \beta \right)}_{f}}\left(\frac{dt}{dy}\sin\omega -\frac{2\alpha_{hnf}\cos\omega }{\alpha_{f}\mathrm{Pe}}\right)=0$$

subject to the boundary conditions of:

(22) $$-{\left.\frac{\partial t}{\partial y}\right|}_{y=0}={\left.\frac{\partial t}{\partial y}\right|}_{y=1}=1.$$

By further integrating, we can determine the temperature distribution in Equation (17). By using Equation (11), we can determine Equations (17) and (18) by integration:

(23) $$\int_{0}^{1}\theta udy=\frac{2\alpha_{hnf}x}{\alpha_{f}\mathrm{Pe}}$$

The entrance to the channel is assumed to be free of heat input. By combining Equations (17) with (23) and using Equation (14), this arbitrary constant is determined by:

(24) $$\int_{0}^{1}t\left(y\right)u\left(y\right)dy=0$$

We can consider the general case of channel inclination (ω>0). The analytical solution, in this case, is as follows:

(25) $$\frac{dt}{dy}=\frac{2\alpha_{hnf}\cos\omega }{\alpha_{f}\mathrm{Pe}\sin\omega }-\left(1+\frac{2\alpha_{hnf}\cos\omega }{\alpha_{f}\mathrm{Pe}\sin\omega }\right)\frac{\sinh\xi \left(1-y\right)}{\sinh\xi }+\left(1-\frac{2\alpha_{hnf}\cos\omega }{\alpha_{f}\mathrm{Pe}\sin\omega }\right)\frac{\sinh\xi y}{\sinh\xi }\;\xi ={\left[2\lambda {\left(1-\varphi_{1}\right)}^{2.5}{\left(1-\varphi_{2}\right)}^{2.5}\frac{{\left(\rho \beta \right)}_{hnf}}{{\left(\rho \beta \right)}_{f}}\sin\omega \right]}^{1/2}>0$$

This velocity distribution is given by:

(26) $$\begin{array}{ll} u\left(y\right)= & \frac{\xi }{2\sinh\xi }\left(1+\frac{2\alpha_{hnf}\cos\omega }{\alpha_{f}\sin\omega }\right)\cosh\xi \left(1-y\right)\\ & +\frac{\xi }{2\sinh\xi }\left(1-\frac{2\alpha_{hnf}\cos\omega }{\alpha_{f}\sin\omega }\right)\cosh\xi y. \end{array}$$

By integrating the expression of Equation (25) and using the condition of Equation (24), we find:

(27) $$\begin{array}{l} t\left(y\right)=\frac{1}{\xi \sinh\xi }\left(1+\frac{2\alpha_{hnf}\cos\omega }{\alpha_{f}\mathrm{Pe}\sin\omega }\right)\cosh\xi \left(1-y\right)+\frac{1}{\xi \sin\xi }\left(1-\frac{2\alpha_{hnf}\cos\omega }{\alpha_{f}\mathrm{Pe}\sin\omega }\right)\cosh\xi y\\ \begin{array}{ccc} & & +\frac{2\alpha_{hnf}\cos\omega }{\alpha_{f}\mathrm{Pe}\sin\omega }Y+c_{1} \end{array}, \end{array}$$

where constant $c_{1}$ is:

(28) $$\begin{array}{l} c_{1}=2{\left(\frac{\alpha_{hnf}\cos\omega }{\alpha_{f}\mathrm{Pe}\sin\omega }\right)}^{2}-\frac{\alpha_{hnf}\cos\omega }{\alpha_{f}\mathrm{Pe}\sin\omega }-\frac{\left(\cosh\xi +1\right)\left(\sinh\xi +\xi \right)}{2\xi \sinh^{2}\xi }\\ \begin{array}{ccc} & & -\frac{2}{\xi } \end{array}{\left(\frac{\alpha_{hnf}\cos\omega }{\alpha_{f}\mathrm{Pe}\sin\omega \sinh\xi }\right)}^{2}\left(2\sinh\xi -\xi \right)\left(\cosh\xi -1\right). \end{array}$$

## 3. Discussion of Results

**Figure 2:**
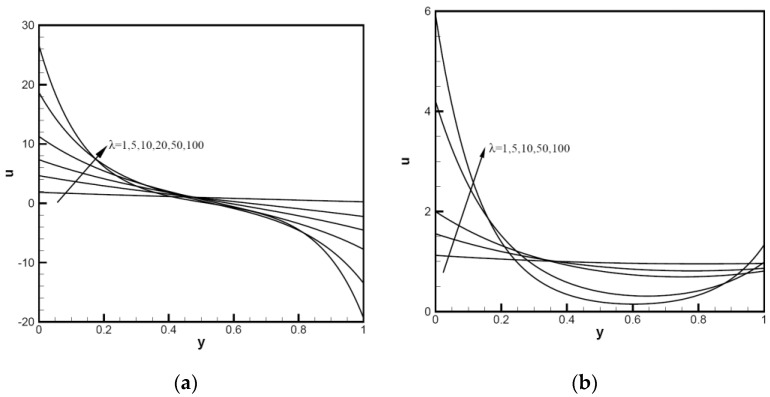
Velocity distributions $u\left(y\right)$ of Cu-Al_2_O_3_-H_2_O hybrid nanofluid: (**a**) with $\varphi_{1}=\varphi_{2}=0.1$, ω=π/6,Pe=1, λ=1,5,10,20,50,100; (**b**) with $\varphi_{1}=\varphi_{2}=0.1$, ω=π/6,Pe=10, *λ* = 1, 5, 10, 20, 50, 100.

**Figure 3:**
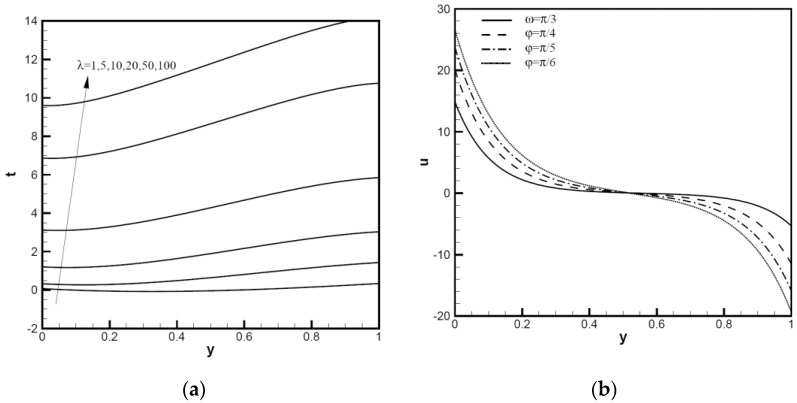
Temperature distributions $t\left(y\right)$ and velocity distributions $u\left(y\right)$ of Cu-Al_2_O_3_-H_2_O hybrid nanofluid: (**a**) with $\varphi_{1}=\varphi_{2}=0.1$, ω=π/6, Pe=1, λ=1,5,10,20,50,100; (**b**) with $\varphi_{1}=\varphi_{2}=0.1$, Pe=1, λ=100, ω=π/6, π/5, π/4, π/3.

The nanoparticle sphericity of Cu-Al_2_O_3_-H_2_O hybrid nanofluid flows between inclined channels filled with a porous medium is investigated. The velocity distribution $u\left(y\right)$ and temperature distribution $t\left(y\right)$ using analytical solutions of different hybrid nanofluids are analyzed and discussed in the following figures; for example, Cu-Al_2_O_3_-H_2_O. It is crucial to understand how the nanoparticle volume fraction and particle sphericity affect the convection performance. The mixed convection parameter λ is used to measure natural (or free) convection effects in comparison to forced convection and Peclet number Pe. We plot the velocity distribution $u\left(y\right)$ and temperature distribution $t\left(y\right)$ in the range of the mixed convection parameter 1≤λ≤100. In Figure 2, considering $u\left(y\right)$ and $t\left(y\right)$ for the tilt angle ω=π/4, the Peclet number is small, and the nanoparticle volume fraction is $\varphi_{1}=\varphi_{2}=0.1$ with Pe=1. For all λ values, except λ=1, the λ value at the upper end of channels indicates a reversed flow, for which Cimpean et al. [30,36] confirmed this behavior of a regular fluid. For λ from 1 to 100, with an increase in the Pe value, the velocity distribution $u\left(y\right)$ with Pe=10 is shown in Figure 2b; the large lambda value near the upper wall has no region of reversed flow. In Figure 3a, the temperature profiles of $t\left(y\right)$ increase significantly between the channel walls as λ increases from 1 to 100. Figure 3b shows the velocity profiles of a Cu-Al_2_O_3_-H_2_O hybrid nanofluid with $\lambda =100,\mathrm{Pe}=1,\varphi_{1}=\varphi_{2}=0.1$, changing with the inclined angle ω. The profiles decrease with the increase in tilt angle ω, and the reversed flows start after the point y=0.5. The smaller the inclined angle of channels to the horizontal direction, the better thermic performance.

**Figure 4:**
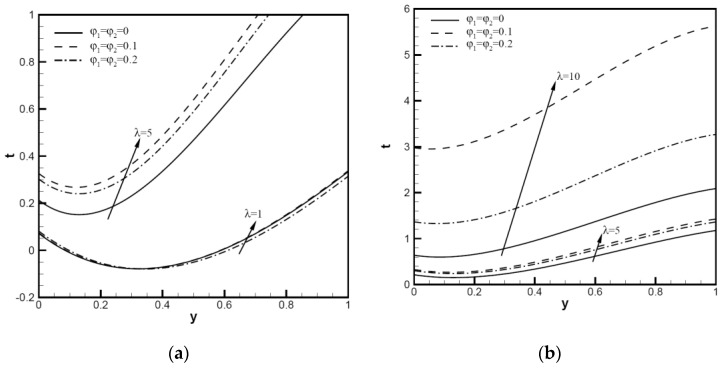
Temperature distributions $t\left(y\right)$ of Cu-Al_2_O_3_-H_2_O hybrid nanofluid: (**a**) with $\varphi_{1}=\varphi_{2}=0,0.1,0.2$, ω=π/6,Pe=1, λ=1,5; (**b**) with $\varphi_{1}=\varphi_{2}=0.1$, ω=π/6,Pe=1, λ=5,10.

**Figure 5:**
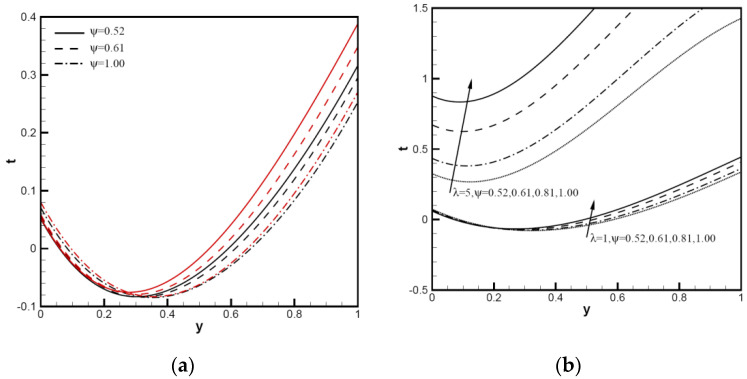
Temperature distributions $t\left(y\right)$ of Cu-Al_2_O_3_-H_2_O hybrid nanofluid: (**a**) with $\varphi_{1}=\varphi_{2}=0.1$ (black), 0.2 (red)ω=π/6,Pe=10, λ=10, ψ=0.52,0.61,1.00; (**b**) with $\varphi_{1}=\varphi_{2}=0.1$, ω=π/6,Pe=1, λ=1,5, ψ=0.52,0.61,1.00.

The temperature distributions of hybrid nanofluids compared with water base fluid are shown in Figure 4. In the case of ω=π/6, Pe=1, λ=1, 5, 10, with an increase in the nanoparticle volume fractions $\varphi_{1},\varphi_{2}$ and mixed convection variable λ, the temperature increases from the bottom wall (y=0) to the upward wall (y=1). The figures show the change of $t\left(y\right)$ with $\varphi_{1}=\varphi_{2}=0.1,0.2$. By adding a small concentration of water, the thermal characteristics of hybrid nanofluids are significantly enhanced. Compared with the water base fluid, the lowest value of temperature distribution moves to the upward wall delayed with the augmentation of λ, and the temperature value decreases in response to an increase in the nanoparticle volume fraction. Hybrid nanofluids have more than doubled thermal performance with an increase in λ. We can thus confirm that the thermal performance has been greatly improved when the fluid contains a few volume fractions of nanoparticles. As shown in Figure 5, the distribution of the temperature for hybrid nanofluids with ω=π/6,Pe=10,λ=10,ψ=0.52,0.61,1.00, $\varphi_{1}=\varphi_{2}=0.1$ (black) and 0.2 (red) was analyzed, respectively. When the nanoparticle volume fraction $\varphi_{1},\varphi_{2}$ increases, the temperature near the bottom plate (y=0) hardly changes, but the temperature near the top plate (y=1) changes significantly. When the nanoparticle sphericity ψ increases, the plate temperature decreases; when the nanoparticle volume fraction increases, the effects of nanoparticle sphericity reduce the increase in the wall temperature. Compared with ψ=0.52, when ψ=0.81 or 1.00, the value of the temperature function $t\left(y\right)$ is relatively close. It can be seen in Figure 4 that when the nanoparticle volume fraction increases from 0 to 0.1, the heat transfer is enhanced. Generally, nanoparticles are uniformly dispersed, and the flow resistance of small particle clusters in the fluid is small, which means that the thermal conductivity is high and the viscosity is relatively low. When the nanoparticle volume fraction increases from 0.1 to 0.2, heat transfer enhancement slows down. Particle content has a direct relationship with Brownian motion intensity and thermal conductivity. At the appropriate mixing ratio, the interaction between particles contributes to thermal conductivity in a much greater way than one nanofluid at the same concentration. The sphericity of nanoparticles has a great influence on thermal conductivity, so it is necessary to further study the influence of the sphericity of nanoparticles.

**Figure 6:**
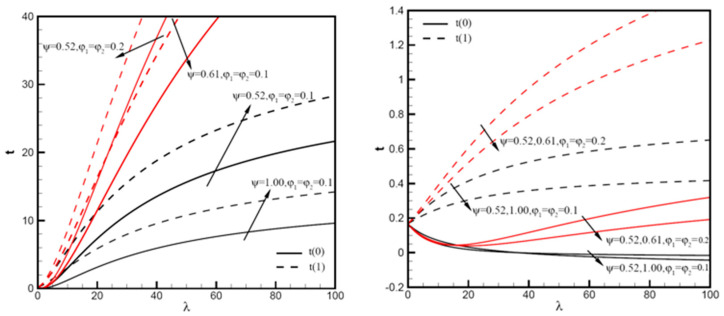
Temperature distributions $t\left(0\right)$ and $t\left(1\right)$ vary with λ of Cu-Al_2_O_3_-H_2_O hybrid nanofluid: (**a**) with $\varphi_{1}=\varphi_{2}=0.1,0.2$, ω=π/6,Pe=1, ψ=0.52,0.61,1.00; (**b**) with $\varphi_{1}=\varphi_{2}=0.1,0.2$, ω=π/6,Pe=10, ψ=0.52,0.61,1.00.

In addition, as shown in Figure 6, profiles $t\left(0\right)$ and $t\left(1\right)$ are determined by mixed convection variable λ with $\varphi_{1}=\varphi_{2}=0.1,0.2$, ω=π/6,Pe=1,10, ψ=0.52,0.61,1.00. In Figure 6a, there is no difference between the curves $t\left(0\right)=t\left(1\right)=0.17$ with Pe=1, and the value of $F\left(1\right)$ enlarges steadily with λ. For Pe=1, curves begin at $t\left(0\right)=t\left(1\right)=0.17$ then enlarge steadily as λ increases. When the volume fractions $\varphi_{1}=\varphi_{2}=0.1,0.2$ are considered, $t\left(0\right)$ begins with a reduction and reaches a minimum value. It is worth noting that we can obtain higher $t\left(0\right)$ and $t\left(1\right)$ values with higher volume fractions. When the nanoparticle sphericity increases, the values of r $t\left(0\right)$ and $t\left(1\right)$ decrease. As shown in Figure 6b, for Pe=10, the value at the beginning of the contour is similar to that of Pe=1 (Figure 6a), and the nanoparticle volume fraction $\varphi_{1},\varphi_{2}$ increases with lambda as well. As a result, $t\left(0\right)=t\left(1\right)=0.17$, and the value of $t\left(0\right)$ decreases as lambda increases. For higher hybrid nanoparticle concentrations, the contour of $t\left(1\right)$ has a very large increase, $\varphi_{1}=\varphi_{2}=0.2$. It can be seen from the figure that the smaller the sphericity of nanoparticles, the stronger the heat transfer. Under the same nanoparticle volume fractions, the smaller the average size of the sphericity of the nanoparticles, the greater the content of small particles, and the larger the specific surface area, so it is easy to form local particle enrichment areas. The particles are arranged more closely inside the liquid, which can reduce liquid layer thickness, and thus the internal heat transfer of nanofluids is enhanced by reducing the thermal resistance between nanoparticles.

## 4. Conclusions

In this paper, the nanoparticle sphericity of Cu-Al_2_O_3_-H_2_O hybrid nanofluid flows is investigated while considering the constant flow rate through the channels as well as the uniform heat flux on wall channels. Analytical solutions are provided for the non-dimensional governing equations. Several parameters are considered in the analytical solutions, including the mixed convection variable, the Peclet number, the channel tilt angle, and nanoparticle sphericity and volume fractions. The results show that effective thermal conductivity increases with an increasing temperature in the same nanoparticle volume fractions. Nanoparticles with a smaller average sphericity size have a greater specific surface area and contain a greater concentration of small particles, which enhances the internal heat transfer of nanofluids. The hybrid nanofluids have suitable stability, and the enhanced heat transfer effect is better with the increase in nanoparticle compositions.

## Figures and Tables

**Table 1:** Water and nanoparticle thermophysics [40,41].

Properties	Cu	TiO_2_	Al_2_O_3_	H_2_O
$C_{p}\left(J/kgK\right)$	385.00	686.20	765.00	4179.00
$\rho \left(kg/m^{3}\right)$	8933.00	4250.00	3970.00	997.10
$\alpha \times 10^{7}\left(m^{2}/s\right)$	1163.10	30.70	131.70	1.47
$k\left(W/mK\right)$	400.00	8.95	40.00	0.61
$\beta \times 10^{-5}\left(1/K\right)$	1.67	0.90	0.85	21.00

## Data Availability

The article contains all the data.
